# Psychological inoculation against climate doom

**DOI:** 10.1073/pnas.2609074123

**Published:** 2026-07-28

**Authors:** Rachel O’Boyle, Tyler Shores, Sander van der Linden

**Affiliations:** ^a^ Department of Psychology, University of Cambridge, Cambridge CB2 3EB, United Kingdom; ^b^ University of Cambridge ThinkLab, Jesus College, University of Cambridge, Jesus Lane, Cambridge CB5 8BL, United Kingdom

**Keywords:** climate change, inoculation, social media, behavior, manipulation

## Abstract

Individuals encounter “climate doom,” extreme portrayals of climate change, on social media. While climate doom may be intended to motivate action, it may also be perceived as manipulative or elicit hopelessness. Yet, no research has examined whether psychological inoculation helps to improve recognition of climate doom or influences engagement with it on social media. In this preregistered study (*n* = 521), we tested whether inoculation improves discernment of climate doom and influences engagement with it by collecting behavioral data using a social media simulator. We find that inoculation substantially improves discernment of climate doom and that this is due to increased perceived manipulation of doom messaging specifically (*d* = 0.68). There was no interaction between the inoculation condition and political leaning and while higher perceived manipulation of doom content predicted lower behavioral intentions, inoculation did not moderate this relationship. Inoculation increased engagement, particularly disliking and flagging of doom content, and decreased sharing. Overall, our results should lead climate communicators to reconsider doom messaging as a mobilization strategy and demonstrate that inoculation is an effective tool for increasing recognition of, and critical engagement with, climate doom across the political spectrum.

News consumption is shifting to social media platforms, with 54% of US adults and 38% of UK adults accessing the news through these platforms (1). Concurrently, 47% of people globally, 39% in the United Kingdom and 35% in the United States encounter climate change news weekly (2), which can prioritize disaster-focused impacts or the direness of the climate situation over opportunity-based reporting (3). This can include ‘climate doom’, extreme portrayals of climate change used to emphasize the threat of global warming. However, climate doom could be strategically used to delay action by convincing people that there is nothing they can do, leading to disengagement and climate inaction (4, 5) or be perceived as emotionally manipulative, undermining the integrity of climate communication (6). Despite extensive research on fear appeals and message persuasiveness (see ref. 7), few studies examine the perceived manipulativeness of such messaging. Existing research suggests that people prefer climate messages without negative emotions (8), that stronger terminology such as “climate emergency” reduces news credibility (9) and that overemphasizing the threat of climate change increases reactance and reduces pro-climate intentions among Republicans (10).

Although there are calls to counter climate doomism (4, 5), no research has examined whether psychological inoculation, i.e., preemptively forewarning people of manipulation and exposing them to weakened dose (examples) of misinformation techniques alongside refutations (11), helps to build resistance against climate doom. Given its emerging effectiveness in other contexts (11), inoculation may improve individuals’ ability to distinguish climate doom from nonmanipulative climate content, hereafter referred to as “standard” climate content. Further, given the potential adverse effects of doom content, improved discernment of climate doom may buffer the relationship between perceived manipulation and lower behavioral intentions.

Moreover, most research in this domain has investigated the impact of climate messages in artificial survey settings unrepresentative of the social media experience. Here we leverage a validated social media feed simulation which collects behavioral engagement data (12). Understanding how inoculation affects online engagement with climate content is important given the prevalence of social media news consumption (1). Whether inoculation increases engagement as individuals spend more time evaluating manipulative content or decreases engagement due to the dismissal of such content is an open question (13–15).

We address these gaps with an experiment. First, we test whether audiences perceive climate doom as more manipulative than standard climate content, and whether perceived manipulation predicts lower pro-environmental intentions. Second, we investigate whether inoculation interacts with political lean to understand whether it has adverse effects particularly among right-leaning audiences. Third, we test whether inoculation influences engagement with climate doom using engagement metrics from a social media simulator (12).

## Study Overview.

**Fig. 1.**
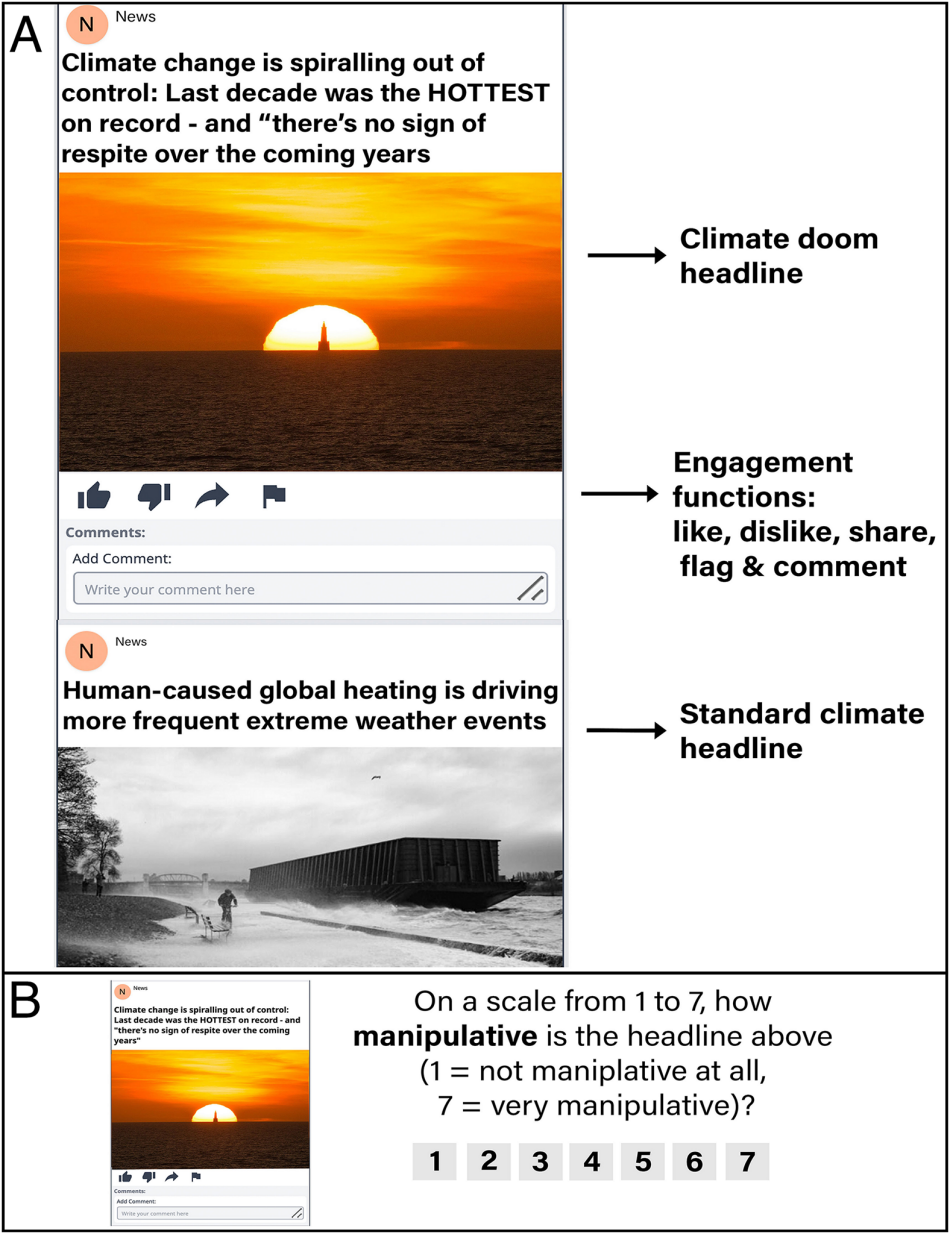
(*A*) Section of the feed which participants scrolled through. (*B*) Manipulation rating task, headlines were randomly presented one by one, with the question below.

Participants from the United Kingdom were randomly assigned to an inoculation or control condition. The treatment contained two components central to cognitive inoculation: participants viewed a short video that 1) forewarned them about emotional manipulation in news headlines and 2) provided them with “microdose” examples of emotional manipulation (e.g., using a sad child to lure people into watching a video). This was followed by relevant climate exemplars (e.g., switching “climate crisis” to “climate apocalypse”), noting how it might make them feel (e.g., hopeless). In the control, participants viewed a drawing video. In both conditions participants answered a comprehension question. Participants were then exposed to a social media simulator (Fig. 1) (12) where we measured engagement (likes, dislikes, shares, comments, and flags) with 12 headlines (four doom, four standard and four unrelated). Last, they rated the perceived manipulativeness of the eight climate-related headlines and reported pro-environmental behavioral intentions (see *SI Appendix* for all measures).

## Results

**Fig. 2.**
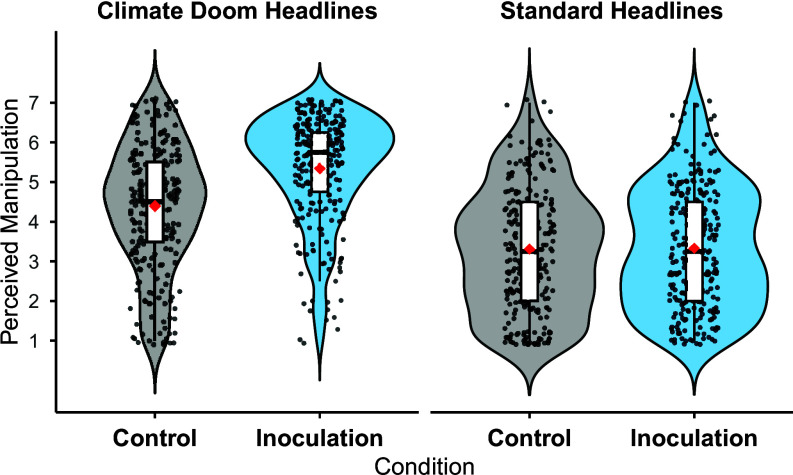
Violin-box plot for perceived manipulation scores across condition and headline. Red diamonds represent the mean; boxes the interquartile range (IQR); the central line the median and; individual observations are shown as jittered points.

To assess whether doom headlines were perceived as more manipulative than standard climate headlines, we conducted separate paired sample *t* tests for each condition (*Ms* and *SDs* in Table 1). Participants rated standard headlines as less manipulative than doom headlines in both the control (t(263)=−14.82, p<0.001, $d_{z}=-0.70$, 95%CI=[−0.88,−0.52]) and inoculation condition (t(256)=−21.33, P<0.001, dz=−1.40, 95%CI=[−1.59,−1.21]). To assess whether inoculation increases manipulation discernment, we conducted a between-subjects Welch’s *t* test. The inoculation condition had significantly and substantially better manipulation discernment than the control, t(486.39)=7.73, P<0.001, d=0.68, 95%CI=[0.50,0.86] (Fig. 2). As each participant rated eight headlines, we also ran a linear mixed-effect model as a robustness check, which aligned with these results (*SI Appendix*).

**Table 1.** Descriptive statistics by condition

	Control		Inoculation	
Variable	*M*	*SD*	*M*	*SD*
Perceived manipulation (1 to 7)				
Standard headlines	3.31	1.55	3.33	1.53
Left-leaning participants	2.71	1.45	2.72	1.42
Right-leaning participants	4.00	1.49	4.07	1.51
Doom headlines	4.40	1.56	5.34	1.34
Left-leaning participants	3.99	1.55	5.18	1.43
Right-leaning participants	4.75	1.59	5.71	1.31
Discernment (standard minus doom)	1.09	1.19	2.01	1.51
Behavioral intentions (1 to 5)	3.28	0.65	3.32	0.63
Total engagement with doom headlines	2.02	2.38	2.24	2.09

To assess whether political lean influenced perceived manipulation, we conducted a two-way ANOVA with condition and political lean (excluding moderates) as the independent variables (IVs) and perceived manipulation of all climate headlines as the dependent variable (DV) (*Ms* and *SDs* in Table 1). The main effect of political lean was significant, such that right-leaning participants rated climate headlines as more manipulative than left-leaning participants (F(1,328)=40.31, P<0.001, $\eta_{p}^{2}=0.11$, 95%CI=[0.05,0.18]). Main effect of condition was also significant (F(1,328)=13.00, P<0.001, $\eta_{p}^{2}=0.04$, 95%CI=[0.008,0.09]) but the interaction was not (F(1,328)= 0.07, P=0.80, $\eta_{p}^{2}=0.0002$, 95%CI=[0.00,0.01]). A linear regression with political lean as a continuous variable including moderates produced similar results (*SI Appendix*).

To assess whether perceived manipulation of doom headlines predicted lower pro-environmental intentions, we conducted a linear regression among the control condition with perceived manipulation of doom headlines as the predictor and behavioral intentions as the DV. Perceived manipulation was a significant negative predictor of behavioral intentions, b=−0.13, 95%CI=[−0.18,−0.08], SE=0.02, t(262)=−5.35, P<0.001. We then tested the full sample including condition and their interaction. Results showed a significant effect of perceived manipulation, b=−0.13, 95%CI=[−0.18,−0.08], SE=0.02, t(517)=−5.37, P<0.001, but not for condition, b=0.12, 95%CI=[−0.01,0.25], SE=0.07, t(517)=1.75, P=0.08, and no interaction, b=0.03, 95%CI=[−0.04,0.11], SE=0.04, t(517)=0.86, P=0.39.

To assess whether inoculation influenced engagement with doom content, we conducted a Mann–Whitney U test (due to violations of normality) which indicated a small but statistically significant difference between groups, U=30535, P=0.04, r=0.10,95%CI=[0.001,0.20]. We also explored whether inoculation influenced each type of engagement by conducting a Mann–Whitney U across each engagement metric. Participants in the inoculation condition disliked ($M_{\text{control}}=0.61$, $SD_{\text{control}}=1.20$, $M_{\text{inoculation}}=1.08$, $SD_{\text{inoculation}}=1.37$) and flagged ($M_{\text{control}}=0.06$, $SD_{\text{control}}=0.30$, $M_{\text{inoculation}}=0.23$, $SD_{\text{inoculation}}=0.66$) doom headlines more than those in the control (dislikes: U=26,420, P<0.001, r=0.22, 95%CI=[0.13,0.31]; flags: U=30,775, P<0.001, r=0.09, 95%CI=[−0.006,0.19]) and shared ($M_{\text{control}}=0.32$, $SD_{\text{control}}=0.84$, $M_{\text{inoculation}}=0.18$, $SD_{\text{inoculation}}=0.58$) doom headlines less than those in the control, (U=36,087, P=0.04, r=−0.06, 95%CI=[−0.16,0.04]). There were no differences for comments (U=35,523, P=0.19) and likes (U=35,490, P=0.24) (*SI Appendix*). We also ran a binomial generalized linear mixed-effects model as a robustness check, which aligned with the results (*SI Appendix*).

## Discussion

We tested whether inoculation—preemptively protecting people against opinion manipulation—increases discernment of climate doom, any potential adverse effects on pro-environmental intentions, and whether inoculation influenced engagement by collecting behavioral data using a social media simulator (12). Inoculation significantly improved participants’ ability to distinguish doom from standard headlines (d=0.68). Critically, this improvement stemmed from increased skepticism toward doom messaging rather than heightened skepticism toward climate change communication overall. In addition, higher perceived manipulation of doom predicted lower behavioral intentions (b=−0.13), aligning with previous research (10). This relationship held regardless of inoculation, indicating that doom messaging, not inoculation, may undermine behavioral intentions. The prevalence of climate doom in the media could thus negatively impact climate action. The absence of an interaction between ideology and condition demonstrates that inoculation improves discernment of climate doom across the political spectrum. Our engagement findings indicate that inoculation increases scrutiny of doom headlines and thus may limit the spread of doom content. Specifically, inoculation increased dislikes (r=0.22) and flags (r=0.09) while decreasing shares (r=−0.06). We note our social media feed comprised 66% climate-related headlines (doom and standard) and 33% unrelated headlines, which is unlikely to be representative of people’s feeds (see *SI Appendix* for full limitations). Future research could explicitly test the influence of different feed content ratios on the inoculation effect. Overall, inoculation can help audiences identify climate doom without backfire effects on behavioral intentions or right-leaning participants. Further, the association between perceived manipulation and lower intentions suggests climate communicators should reconsider climate doom as a mobilization strategy.

## Materials and Methods

This study was preregistered on AsPredicted and approved by the Cambridge Psychology Research Ethics Committee (PRE.2025.051). A quota sample of UK adults based on sex, age, and political party affiliation was recruited through Prolific (n=521) who provided informed consent. The study was a mixed design. The within-subject variable was headline type: doom and standard. The between-subjects variable was condition: control (n=264) and inoculation (n=257). To measure perceived manipulation for each headline, we asked participants: “On a scale from 1 to 7, how manipulative is the headline above?” (1 = not manipulative at all, 7 = very manipulative). To measure pro-environmental intentions, we asked: “How often do you intend to take the following actions which can help to reduce your individual carbon emissions?” for nine items on a 5-point scale from never to always (M=3.30, SD=0.64, Cronbach’s alpha = 0.76). Engagement was the total number of times a participant liked, disliked, shared, commented on or flagged doom headlines. To measure political lean we asked: “On the scale below, please indicate where you place yourself on the political spectrum (very left-wing, left-wing, moderate, right-wing, very right-wing).” Participants were grouped into: left ($n_{\text{total}}=231$, $n_{\text{control}}=117$, $n_{\text{inoculation}}=114$), moderate ($n_{\text{total}}=189$, $n_{\text{control}}=92$, $n_{\text{inoculation}}=97$) and right ($n_{\text{total}}=101$, $n_{\text{control}}=55$, $n_{\text{inoculation}}=46$). See *SI Appendix* for additional methodological details, measures, preregistered analyses, robustness checks and power analyses. The authors declare the use of Claude Sonnet 4.6 and ChatGPT 5 for checking code and proofreading text.

## Supplementary Material

Appendix 01 (PDF)

## Data Availability

Data, code, preregistration, and QSF are deposited on OSF (https://osf.io/74cgf) (16).
