# Supplementary material for: Psychological inoculation against climate doom
Source: Proc Natl Acad Sci U S A. 2026 Jul 28;123(31):e2609074123. doi: 10.1073/pnas.2609074123 (PMC13438516; doi:10.1073/pnas.2609074123)
Supplement: Supplementary file 1 [file pnas.2609074123.sapp.pdf]

Supporting Information for

Psychological Inoculation Against Climate Doom

Rachel O'Boyle, Tyler Shores and Sander van der Linden

Rachel O'Boyle or Sander van der Linden
E-mail: rachel-oboyle@outlook.com or sv395@cam.ac.uk

This PDF file includes:

Supporting text
SI References

Supporting Information Text

Extended Methods.

A. Participants. A quota sample of UK adults based on sex, age and political party affiliation of 521 participants were recruited through Prolific (51% female, age range 18 - 81, $M_{age} = 46$, 44.33% left leaning, 36.28% moderate; 19.38% right leaning; 43.60% see climate change news on social media a few times per week). The quotas were Prolific's default UK political representative sample stratified by: age (using five brackets: 18-24, 25-34, 35-44, 45-54 and 55+); 'Sex' (male and female); political affiliation' (Conservative, Labour, Liberal Democrats, Green, SNP, Reform and other). Prolific based political affiliation targets on 2024 data from YouGov (1) and sex, ethnicity age from Census data from the Office for National Statistics (e.g. (2)). For a full breakdown of our sample statistics please see Table 1 in 'Supplementary Descriptives and Analysis' on OSF (3). All participants provided informed consent and were compensated for their time. As per the pre-registration participants who failed two attention checks ($n = 0$) or were using a Safari browser ($n = 28$) were screened out of the survey due to incompatibility with social media simulator embedded in Qualtrics (e.g. redirected and asked to return the survey) and those who scored less than 0.5 on ReCaptcha ($n = 44$) and failed either attention check ($n = 0$) completed the survey but were excluded from analysis.

B. Measures.

B.1 Manipulation. Participants were asked on a 7-point Likert scale, "On a scale from 1 to 7, how manipulative is the headline above (1 = not manipulative at all, 7 = very manipulative)?" ($M_{\text{doom}} = 4.86$, $SD_{\text{doom}} = 1.53$, $\text{IQR}_{\text{doom}} = 2$, $M_{\text{standard}} = 3.32$, $SD_{\text{standard}} = 1.54$, $\text{IQR}_{\text{standard}} = 2.5$).

B.2 Behavioural Intentions. Participants were asked "How often do you intend to take the following actions which can help to reduce your individual carbon emissions?" on a 5-point scale from never to always ($M = 3.30$, $SD = 0.64$, $Cronbach'salpha = .76$) for nine behavioural intention items: 'Recycling', 'Buying fewer things', 'Reducing food waste', 'Eating a vegetarian diet', 'Eating a vegan diet', 'Avoiding one long-haul flight', 'Turning down the heating', 'Living car-free', 'Using energy-efficient appliances'.

B.3 Engagement. We collected several engagement metrics for each post including: likes, dislikes, flags, shares and comments. For each participant, we calculated total engagement across all metrics with each headline type (i.e. doom, standard and unrelated) as well as the total number of times a participant liked, disliked, flagged, shared or commented for each headline type. For example, there were four posts per headline type so a participant's total like score per headline type would be within a range of 0 to 4. For total engagement, doom headlines averaged 2.13 per participant ($SD = 2.24$, range = 10), standard headlines averaged 2.10 per participant ($SD = 2.20$, range = 12), and unrelated headlines averaged 2.32 per participant ($SD = 1.90$, range = 9). For likes, doom headlines averaged 0.50 per participant ($SD = 1.00$, range = 4), standard headlines averaged 0.67 per participant ($SD = 1.16$, range = 4), and unrelated headlines averaged 1.44 per participant ($SD = 1.18$, range = 4). For dislikes, doom headlines averaged 0.84 per participant ($SD = 1.31$, range = 4), standard headlines averaged 0.64 per participant ($SD = 1.10$, range = 4), and unrelated headlines averaged 0.32 per participant ($SD = 0.61$, range = 4). For shares, doom headlines averaged 0.25 per participant ($SD = 0.73$, range = 4), standard headlines averaged 0.35 per participant ($SD = 0.84$, range = 4), and unrelated headlines averaged 0.13 per participant ($SD = 0.41$, range = 3). For flags, doom headlines averaged 0.15 per participant ($SD = 0.52$, range = 4), standard headlines averaged 0.05 per participant ($SD = 0.30$, range = 3), and unrelated headlines averaged 0.02 per participant ($SD = 0.17$, range = 3). For comments, doom headlines averaged 0.39 per participant ($SD = 0.90$, range = 4), standard headlines averaged 0.40 per participant ($SD = 0.88$, range = 4), and unrelated headlines averaged 0.39 per participant ($SD = 0.82$; range = 4). See Table 2 in 'Supplementary Descriptives and Analysis' on OSF (3) for means, *SDs* and IQRs for each engagement metrics per headline type.

B.4 Climate Change Beliefs. Participants were asked four questions to measure their general climate change beliefs ($M = 5.38$, $SD = 1.21$, $Cronbach'salpha = .79$) on a 7-point Likert scale (1= strongly disagree, 7 = strongly agree): Climate change is a proven fact (real); There is much uncertainty and no scientific consensus about the true causes of climate change (human-caused); Climate change is on track to cause notable human suffering (e.g. mass migration or armed conflict about resources) (threat) and; Fighting climate change can actually create new jobs, economic opportunities and growth (mitigation) (4). The second item (human-caused) was reverse scored.

B.5 Fatalism and Efficacy Beliefs. Participants were asked three questions on a 7-point Likert scale from strongly disagree to strongly agree to measure: fatalism ("It's already too late to do anything about climate change" (5) ($M = 2.51$, $SD = 1.43$, range = 6); individual efficacy "My personal actions won't make any difference to climate change" ($M = 3.15$, $SD = 1.76$, range = 6); and collective efficacy: "Collective action won't make any difference to climate change" ($M = 2.26$, $SD = 1.49$, range = 6).

B.6 Comprehension Check. Participants in the inoculation condition took part in a comprehension check in which they were asked to identify which headline contained fear mongering. 90.27% of participants in the inoculation condition correctly identified the misleading headline after viewing the inoculation video. Participants in the control condition took part in a comprehension check related to the control video on how to draw a face (1.89% failed the quiz in the control). See "Materials"

on OSF (3) for screenshots of the comprehension check for each condition. As per the pre-registration we re-ran the analysis excluding those who failed the quiz ($n = 30$). All the results aligned with the main paper apart from the significant difference of the non-parametric test for total/overall engagement which was close but technically no longer significant ($W = 27,518$, $p = .10$, $r = .08$, $95\%CI = -0.02, 0.19$) and the generalised binomial mixed effect model for flags was technically no longer significant ($OR : 3.54$, $95\%CI = 0.87 - 14.1$, $SE = 2.50$, $z = 1.79$, $p = .07$).

B.7 Attention Checks. Two attention checks were included. In the first, participants were asked: “The following question is to verify that you are paying attention. Please select all five answers below.”. In the second, participants were asked: “The following question is to verify that you are paying attention. Please type purple in the box below.”.

B.8 Demographic Variables and Media Use. Participants were asked several demographic questions including age (age in years), gender (male, female, non-binary, other, prefer not to say), education (No formal education above age 16, Professional or technical qualifications above age 16, School education up to age 18, Bachelor’s degree or equivalent, Master’s degree or other postgraduate qualification, Doctorate), political lean (very left-wing, left-wing, moderate, right-wing, very right-wing). Participants were also asked about their media use including frequency of news checking, social media use and seeing climate change related news in the media (A few times a day, Everyday, A few times a week, Once a week, Once a month and Never).

B.9 Manipulation Discernment. Manipulation discernment was calculated by taking the average perceived manipulativeness of doom headlines minus the average perceived manipulativeness of standard headlines ($M = 1.54$, $SD = 1.43$, $IQR = 2.00$).

B.10 Perceived Manipulation of Climate Headlines. Perceived manipulation of climate related headlines was calculated by taking average manipulation score of standard and doom content ($M = 4.09$, $SD = 1.35$, $IQR = 1.75$).

C. Materials.

C.1 Headlines. There were three types of headlines: climate doom headlines, standard climate headlines and unrelated headlines. Headlines were adapted from actual news headlines posted online by The Guardian, BBC and the Daily Mail to represent a range of news articles from left, centre and right leaning news outlets. Standard headlines were selected from news headlines in the media that are negative in tone but convey objective facts without any emotional manipulation. The majority of headlines remained unchanged, several were slightly adapted to add language intensifiers to doom headlines or remove language intensifiers from standard headlines (see ‘Materials’ on OSF (3) for all headlines).

C.2 Simulated Social Media Platform. A social media simulator was developed using ‘The Misinformation Game’ (created by (6)). The simulator had the following components: welcome, instruction and prompt screens before the feed; a feed which consisted of posts participants can scroll through and engage with by liking, disliking, flagging, sharing or commenting and; a debrief screen. For the purpose of this study, the feed contained twelve posts (four climate doom, four climate standard and four unrelated to climate change). Each post had no social cues (e.g. likes, dislikes) and the same "News" source to ensure any differences could be attributed to the condition. The order of all stimuli was randomised. The platform was called ‘Social Sphere’. Please see ‘Materials’ on OSF (3) for screenshots of each page of the social media simulator.

C.3 Inoculation. The inoculation consisted of a 30 second emotional manipulation inoculation video from (7), followed by a short piece of text on how this relates to climate change and a comprehension check to test whether participants can identify emotional manipulation. If participants got the comprehension check right, they were presented with ‘You’re correct’ screen, if they got it wrong they were presented with the ‘Not quite right’ screen. The control condition watched a 30 second video on how to draw a face followed by a quiz question on the video (8). Please see the ‘Materials’ file on OSF (3) for screenshots of the comprehension check and the text on how inoculation relates to climate change.

D. Procedure. Participants were recruited from Prolific, redirected to Qualtrics where the experiment was hosted, and provided with an information sheet and consent form. They were asked whether they were using a Safari browser, if they were, they were screened out because the timing function required for social media simulator section is not compatible with Safari browsers. If they provided consent, they completed a ReCaptcha and attention check. Participants were randomly assigned to one of the two conditions: control or inoculation. Participants in the inoculation condition were presented with the inoculation procedure described in C.3 and participants in the control condition were presented with the control procedure also described in C.3. Before exposure to the social media simulator feed, participants received instructions on how to use the social media simulator (e.g. how to use the comment function) and were prompted to interact with the social media simulator as they normally would on social media. On the social media simulator participants were exposed to all doom, standard and unrelated headlines. After interacting with the platform participants were taken to a new screen and given completion code which they were required to input into a follow up question to confirm that they had interacted with the social media simulator. Participants were not able to move onto follow up questions until they had reached this page to ensure that they did not skip through this part of the experiment. Next, participants completed a second attention check before proceeding to the pro-environmental behavioural intention questions. Then they were exposed to the doom headlines and standard headlines in a random order, and underneath each headline asked to rate perceived manipulation. Then participants answered climate change belief, fatalism, efficacy, demographic and media use questions. Lastly, the participants were debriefed.

E. Balance Checks. To check conditions were balanced, we used Pearson's chi-square tests for key demographic and political variables. No statistically significant differences were observed between conditions for gender, $X^2(4) = 7.30$, $p = .12$, or political lean, $X^2(4) = 3.78$, $p = .44$. However, a significant difference was detected for education, $X^2(5) = 14.35$, $p = .01$.

F. Engagement.

F.1 Full Likes and Comments Results. A Mann-Whitney U test indicated that there was no difference in likes ($M_{control} = 0.57$, $SD_{control} = 1.09$, $M_{inoculation} = 0.43$, $SD_{inoculation} = 0.89$) and comments ($M_{control} = 0.45$, $SD_{control} = 0.99$, $M_{inoculation} = 0.32$, $SD_{inoculation} = 0.79$) between the conditions (likes: $U = 35489.50$, $p = .24$, $r = .05$, 95% $CI = [-0.05, 0.14]$; comments: $U = 35523$, $p = .19$, $r = .05$, 95% $CI = [-0.05, 0.15]$).

F.2 Mediation Analysis. As per the pre-registration, we analysed whether perceived manipulation mediates the relationship between condition and engagement. Mediation was tested using a causal mediation analysis with bootstrapped confidence intervals to provide a more accurate mediation test without assuming normality. All results were not significant. The total effect of condition on engagement was 0.23, 95% $CI = [-0.16, 0.61]$, $p = .26$. The ACME was 0.02, 95% $CI = [-0.10, 0.16]$, $p = .77$. The ADE was 0.21, 95% $CI = [-0.21, 0.62]$, $p = .33$. The proportion of the total effect mediated was 0.08, 95% $CI = [-2.81, 2.40]$, $p = .84$.

G. Power Analyses. We conducted power analysis using G* power to identify the minimum effect size our study could reliably detect. This indicated that the available sample size with 95% power at a significance level of 0.05 was sufficient to detect the following effect sizes: $dz = 0.21$ for a within-subject t-test with a sample size 257 participants; $d = 0.29$ for a between subject t-test with the full sample; $f^2 = 0.05$ for a linear regression within the control with a sample size 264 participants; $f^2 = 0.03$ for a moderation analysis with the full sample; $f^2 = 0.2$ for a two-way ANOVA with moderates removed ($n = 332$) and $d = 0.32$ for a Mann-Whitney U test with the full sample.

H. Robustness Checks.

H.1 Comparing perceived manipulation within each condition. A Shapiro-Wilk test showed significant deviations from normality across standard and doom perceived manipulation scores for both the control and inoculation condition (all p <.001). Visual inspections of histograms and QQ plots revealed minor to moderate skews. Therefore, a Wilcoxon signed-rank test was conducted as a robustness check to compare perceived manipulation scores between standard and doom headlines within the control and inoculation condition. The test indicated that perceived manipulation scores were significantly lower for the standard headlines than for the doom headlines in both conditions (control: $V = 1502$, $W = 24606$, $Z = 11.58$, $p < .001$, matched rank biserial correlation = .88, 95%$CI = [-0.91, -0.85]$; inoculation: $V = 454.5$, $W = 28{,}948.50$, $Z = 13.07$, $p < .001$, matched rank biserial correlation = .97, 95% $CI = [-0.98, -0.96]$).

H.2 Linear mixed effects model for perceived manipulation. Given that each participant was exposed to (and rated) eight headlines, four standard and four doom, we ran a linear mixed-effects model to examine the effects of condition and headline type (standard versus doom) on perceived manipulation including random intercepts for both participants and each post. The control condition and doom headlines were used as reference groups. There was a significant main effect of condition ($b = 0.94$, 95%$CI = [0.70, 1.18]$, $SE = 0.12$, $t(638.77) = 7.64$, $p < .001$), indicating that the inoculation condition perceived doom headlines as more manipulative than the control. There was a significant main effect of headline type ($b = -1.09$, 95%$CI = [-1.78, -0.40]$, $SE = 0.36$, $t(6.14) = -3.05$, $p = .02$), indicating that standard headlines were rated as less manipulative than doom headlines. There was a significant interaction between condition and headline type ($b = -0.92$, 95%$CI = [-1.07, -0.77]$, $SE = 0.08$, $t(3639.00) = -11.88$, $p < .001$), such that inoculation increased perceived manipulation of doom headlines but had a negligible effect on standard headlines. Random effects for participants indicated substantial variation ($\sigma^2 = 1.58$, $SD = 1.26$). These results align with the t-tests reported in the text in the main paper.

H.3 Examining the effects of political lean.

Additional multiple linear regression testing the influence of political lean on perceived manipulation of all climate content including moderates (using a 5-point continuous political lean scale. We fitted a multiple linear regression model with political lean (as a continuous variable and centered to midpoint), condition and their interaction as predictors, and perceived manipulation of all climate-related headlines as the DV. Condition was a significant positive predictor of perceived manipulation of climate-related headlines ($b = 0.48$, 95%$CI = [0.25, 0.71]$, $t(517) = 4.06$, $p < .001$). Political lean was also a significant positive predictor ($b = 0.55$, 95%$CI = [0.37, 0.72]$, $t(517) = 6.21$, $p < .001$), such that right-lean positively predicts perceived manipulation of climate-related headlines. The interaction between condition and political lean was not statistically significant ($b = -0.12$, 95%$CI = [-0.37, 0.14]$, $t(517) = -0.91$, $p = .36$), suggesting that the relationship between political lean and perceived manipulation of climate-related headlines did not differ significantly across condition.

Re-run of analysis using a re-coded political lean variable based on Prolific party affiliation data. We re-ran the two-way ANOVA reported in main text using Prolific's political party affiliation variable, re-coded into three Prolific-based political lean groups: right (conservatives, UKIP and Reform UK), moderate (Liberal Democrats) and left (Labour, Green Party and Scottish National Party (SNP)). As per the original ANOVA, we excluded moderates. The main effect Prolific-based political lean was significant, such that right-leaning participants rated climate-related headlines as more manipulative than left-leaning participants ($F(1,446) = 33.80$, $p < .001$, $\eta_p^2 = .07$, $95\%CI = [0.03, 0.12]$). Main effect of condition was also significant ($F(1,446) = 13.17$, $p < .001$, $\eta_p^2 = .03$, $95\%CI = [0.006, 0.07]$) but the interaction was not ($F(1,446) = 1.02$, $p = .31$, $\eta_p^2 = .0002$, $95\%CI = [0.00, 0.02]$).

Both robustness checks indicate that inoculation increases perceived manipulation of all climate-related headlines and that right-leaning participants are more likely to perceive climate-related headlines as more manipulative than left-leaning participants but the effect of inoculation does not differ across left-and right-leaning participants. Given the minimal change in perceived manipulation of standard headlines (see Table 1 and results reported in the main paper), we can infer that increases in perceived manipulation are driven by increases in perceived manipulation of doom headlines.

H.4 Binomial generalised linear mixed-effects model for engagement metrics. Given that each participant was exposed to (and rated) eight headlines (four standard and four doom), we ran binomial generalised linear mixed-effects models across all types of engagement metric. The models examined the effects of condition and headline type (standard versus doom) for each engagement metric including random intercepts for participants. The control condition and doom headlines were used as reference groups. We did not include post as a random intercept because preliminary testing showed the post variance to be negligible (e.g. 0.021 for likes). Two participants were removed from analysis because they had missing engagement data and we ran a zero-inflated model for sharing because it had a better fit (see Section "S.A.2" in the 'Supplementary Descriptives and Analysis' on OSF (3) for a full table of results and assumption checks). Please note that we did not include dwell time per post because it is deemed as unreliable by the developers of the social media simulator but we report the descriptives for total time spent on the platform here: $M_{control} = 575$, $SD = 258$; $M_{inoculation} = 605$, $SD = 262$.

The results aligned with the Mann-Whitney U tests, indicating that participants in the inoculation condition had a higher probability of disliking and flagging, and a lower probablity of sharing. The additional interaction term revealed that participants within the inoculation condition also had a lower likelihood of liking and sharing and a higher likelihood of disliking and flagging doom headlines compared to standard headlines (see below). The large participant variance in all models indicates substantial individual differences in engagement behaviour (likes: $\sigma^2 = 8.92$, $SD = 2.99$; dislikes: $\sigma^2 = 7.62$, $SD = 2.76$; shares: $\sigma^2 = 93.10$, $SD = 9.64$; flags: $\sigma^2 = 34.67$, $SD = 5.89$; comments: $\sigma^2 = 17.65$, $SD = 4.20$), especially for sharing.

Likes: Neither condition ($OR = 0.66$, $95\%CI = [0.33 - 1.32]$, $SE = 0.24$, $z = 1.18$, $p = .24$) nor headline type ($OR = 1.10$, $95\%CI = [0.79 - 1.54]$, $SE = 0.19$, $z = 0.59$, $p = 0.55$) were significant. However, there was a significant interaction between condition and headline type ($OR = 2.62$, $95\%CI = [1.63 - 4.20]$, $SE = 0.63$, $z = 3.99$, $p < .001$). Specifically, participants in the inoculation condition had higher probabilities of liking standard headlines compared to doom headlines.

Dislikes: There was a significant main effect of condition ($OR = 5.08$, $95\%CI = [2.71 - 9.54]$, $SE = 1.63$, $z = 5.06$, $p < .001$) and a significant interaction between condition and headline type ($OR = 0.33$, $95\%CI = [0.21 - 0.51]$, $SE = 0.07$, $z =$ -4.95, $p < .001$). There was no significant main effect of headline type ($OR = 1.03$, $95\%CI = [0.74 - 1.43]$, $SE = 0.17$, $z = 0.17$, $p = .87$).

Shares: There was a significant main effect of condition ($OR = 0.10$, $95\%CI = [0.02 - 0.40]$, $SE = 0.07$, $z =$ -3.30, $p < .001$) and a significant interaction between condition and headline type ($OR = 11.80$, $95\%CI = [3.97 - 34.90]$, $SE = 6.53$, $z = 4.45$, $p < .001$). Headline type was not significant ($OR = 1.98$, $95\%CI = [0.83 - 4.73]$, $SE = 0.88$, $z = 1.54$, $p = 0.18$).

Flags: There was a significant main effect of condition ($OR = 3.98$, $95\%CI = [1.06 - 15.00]$, $SE = 2.69$, $z = 2.05$, $p = .04$) and a significant interaction between condition and headline ($OR = 0.24$, $95\%CI = [0.07 - 0.79]$, $SE = 0.15$, $z =$ -2.34, $p = 0.02$). There was no significant main effect of headline type ($OR = 0.49$, $95\%CI = [0.19 - 1.30]$, $SE = 0.24$, $z =$ -1.43, $p = 0.16$).

Comments: There was no significant main effect of condition ($OR = 0.62$, $95\%CI = [0.27 - 1.42]$, $SE = 0.26$, $z =$ -1.13, $p = 0.26$), headline type ($OR = 0.93$, $95\%CI = [0.63 - 1.35]$, $SE = 0.18$, $z =$ -0.39, $p = .70$) or significant interaction between condition and headline type ($OR = 1.36$, $95\%CI = [0.78 - 2.35]$, $SE = 0.38$, $z = 1.08$, $p = 0.28$).

H.5 Testing whether perceived manipulation of doom content, condition, and their interaction predicted behavioural intentions. Given education was unbalanced, we ran a linear regression model examining the effects of perceived manipulation in doom headlines, condition, and their interaction on behavioural intentions, controlling for education. Perceived manipulation was a significant negative predictor of behavioural intentions, $b = -0.13$, 95% $CI = [-0.18, -0.08]$, $SE = 0.02$, $t = -5.36$, $p < .001$, indicating that higher perceived manipulation was associated with lower behavioural intentions. Neither the main effect of condition, $b = 0.12$, 95% $CI = [-0.02, 0.25]$, $SE = 0.07$, $t = 1.73$, $p = .08$, nor the interaction between perceived manipulation and condition was statistically significant, $b = 0.03$, 95% $CI = [-0.04, 0.11]$, $SE = 0.04$, $t = 0.85$, $p = .39$.

I. Pre-registration. Our pre-registration can be found on OSF (3) (https://osf.io/74cgf). Due to the limited length of the brief report format, we could not report all of our pre-registered analyses in the main paper so this can be found in 'Supplementary Descriptives and Analysis' on OSF (3).

J. Limitations. This study is not without limitations. First, the outcome measures may not translate into real world behaviour. In particular, behavioural intentions do not necessarily predict actual behaviour, and while one aim of the study was to approximate real-world social media engagement through the use of a simulated platform, engagement may nevertheless be overestimated or underestimated relative to naturalistic settings. Second, the ecological validity of the social media simulation remains limited. Although 'noise' was introduced by incorporating non–climate-related posts, these constituted only 33% of the feed, which does not accurately reflect the proportion of climate content individuals encounter during typical scrolling sessions. In addition, the platform was not personalised, further constraining the realism of the simulated social media experience. Third, while perceived manipulation was a significant negative predictor of pro-environmental behavioural intentions, we cannot determine whether perceiving content as manipulative reduces motivation to act. Future experimental work could test causation. Fourth, the sample was based on Prolific's representative political sample, which at the time of data collection was calibrated using a YouGov poll conducted in 2024 (1). This distribution may not fully reflect the current UK distribution of political party affiliation. Specifically, the sample contains a higher proportion of Labour-identifying (left-leaning) participants and a lower proportion of Reform UK-identifying (right-leaning) participants than would be expected based on current population estimates. Fifth, participants using a Safari browser were screened out due to incompatibility with social media simulator embedded in Qualtrics. Although, we suggested using Google Chrome or another browser, if Safari users chose not to take part are findings could be less representative of Macbook users.

References

1. YouGov, How Britain voted in the 2024 general election. (2024).
2. Office for National Statistics, Rm032 - ethnic group by sex by age (2021) https://www.nomisweb.co.uk/datasets/c2021rm032.
3. R. O'Boyle, T. Shores, S. van der Linden, Psychological inoculation against climate doom (OSF) (2026) . https://osf.io/74cgf. Deposited 9 June 2026.
4. B. E. Hilbig, Judgments and beliefs about climate change: Measurement, stability, and behavioral consequences. *Judgm. Decis. Mak.* **19** (2024).
5. J. R. Marlon, et al., How hope and doubt affect climate change mobilizationr. *Front. Commun.* **4** (2019).
6. L. H. Butler, et al., The (mis)information game: A social media simulator. *Behav. Res. Methods* **56**, 2376–2397 (2024).
7. R. Maertens, et al., The long-term effectiveness of video-based inoculation: Three longitudinal experiments (OSF) (2022) . https://osf.io/d736c.
8. B. Shearer, How to draw a face: 30 SECOND ART LESSON (beginner tutorial) (YouTube) (2021) [Video recording].
